# Postextraction sinus lining prolapse

**DOI:** 10.1002/ccr3.3505

**Published:** 2020-11-06

**Authors:** Dennis Flanagan

**Affiliations:** ^1^ Connecticut Dental Groups Willimantic CT USA

**Keywords:** antrum, buccal fat pad, decongestant, herniation, prolapse

## Abstract

A prolapse of the sinus lining through an extraction socket occurred after 4 weeks. The lining was pushed into the socket and maintained with a buccal fat pad pedicle graft. Healing was complete after several weeks. A sinus decongestant can be prescribed to promote sinus drainage and reduce pressure.

## Graphical abstract

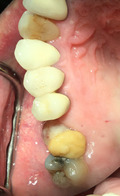

## 1 INTRODUCTION

A herniation or prolapse of the sinus lining through an extraction socket is a rare occurrence but can be surgically managed. Four weeks subsequent to a maxillary molar tooth extraction, a prolapse or herniation of the antrum lining is herein reported. The prolapsed lining was disinfected, repositioned back up into the extraction socket and held in place with collagen plugs and covered with a fat pad pedicle graft and silk sutures. After 4 weeks, healing was almost complete. After 7 weeks of healing, the site was completely closed with no oral‐antral communication or fistula. Postoperative instructions for maxillary molar extractions should include sinus care for nose blowing and sneezing. To reduce the risk for sinus complications, a sinus decongestant can be instituted to ensure the drainage of the ostium. Intra‐antral pressure may promote an occurrence of a herniated sinus lining. While a rare complication, a postextraction sinus lining prolapse can be corrected with a buccal fat pad pedicle graft.

Extraction of a posterior maxillary tooth is a routine procedure for most dentists. A protrusion or herniation of the sinus lining into and through the postextraction socket is unusual and rare. Nonetheless, this may occur and potentially develop into a fistula.^1^, ^2^ Herniation of the antral lining through a recent extraction socket may be misdiagnosed as a neoplasm.^1^, ^2^

## 2 CASE REPORT

**FIGURE 1:**
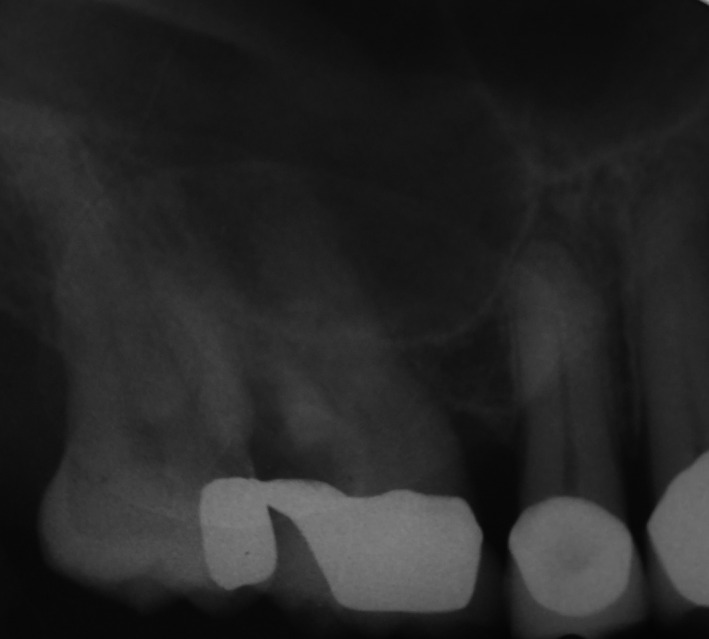
Preoperative radiograph of the unrestorable maxillary right first molar tooth

A 67‐year‐old retired pharmacist with an unremarkable medical history presented for treatment of a “fractured tooth” (Figure 1). The maxillary right second molar crowned tooth had drifted into the site of the previously extracted first molar. It had sustained distal caries that weakened the mesiodistal and palatal roots and was deemed unrestorable. The proximity of the antrum and radiographic opacification was noted on the periapical radiograph (Figure 1). After local anesthesia (1.6cc articaine 4%, Septocaine), the tooth was sectioned using an air‐driven surgical handpiece and #558 surgical burrs. The roots were carefully removed, and the socket inspected and carefully debrided. No sinus perforation was found. A collagen plug was placed in the socket and retained with 3‐0 chromic suture. Since no sinus lining perforation was found, no sinus instructions were instituted.

**FIGURE 2:**
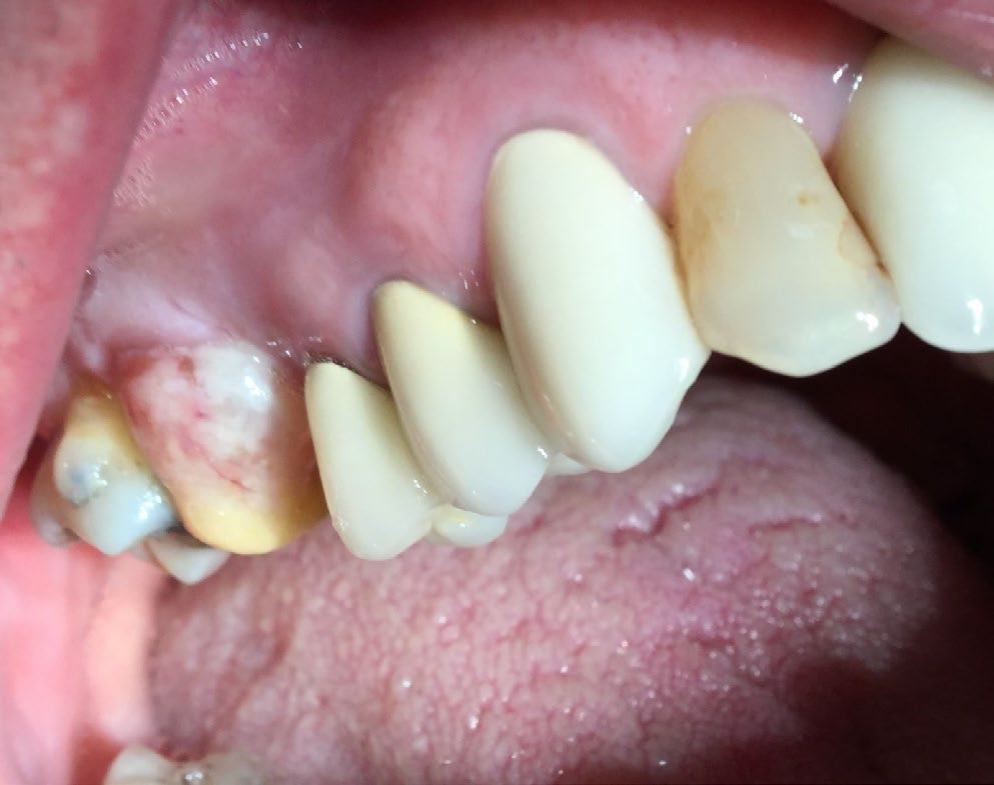
After four postoperative weeks, the patient complained of a yellow protrusion that he was able to push back into the socket but would not stay in place

**FIGURE 3:**
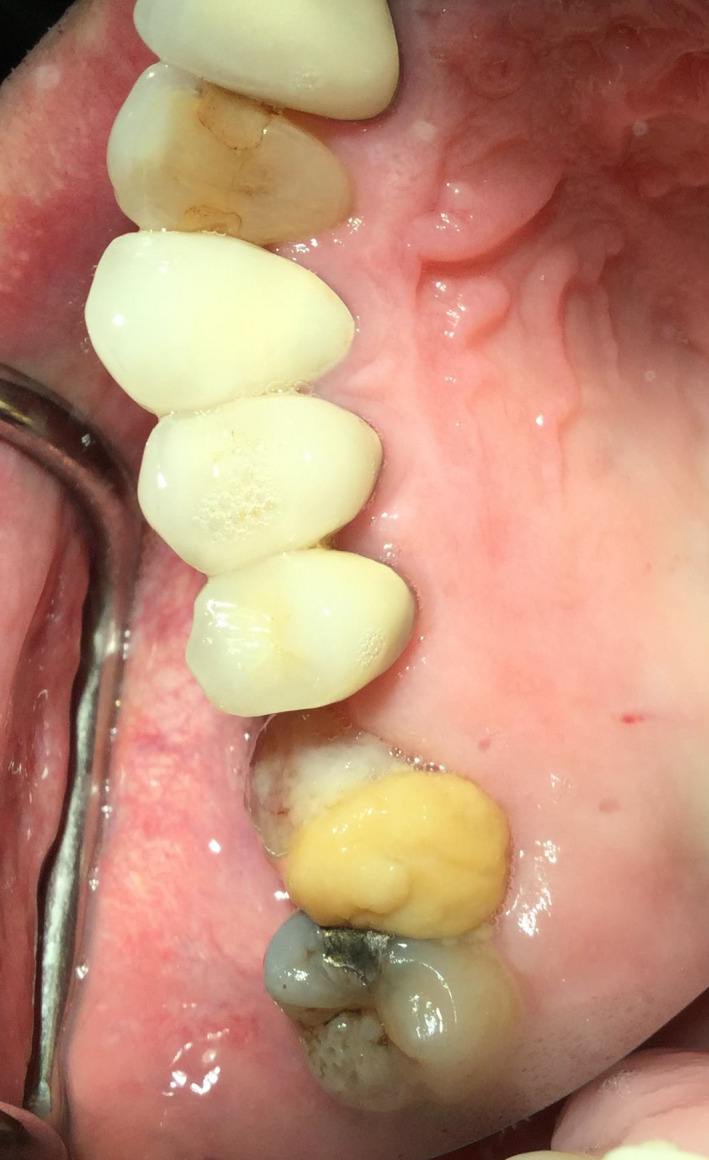
The prolapsed lining was pushed superiorly into the socket to the perceived level of the sinus floor and held with two collagen plugs

After 4 weeks, the patient presented with a complaint of a soft yellow protrusion that he was able to push back into the healing socket but would not stay in place (Figures 2 and 3). Upon examination, the red and yellow protrusion was pushed up into the socket but immediately again protruded with respiration.

There was no pain or bleeding nor airflow communication with the sinus. The lesion appeared to be an obvious prolapse or herniation of the sinus lining. Cutting into the prolapsed lining or biopsy was deemed contraindicated due the risk for an oral‐antral fistula formation.

**FIGURE 4:**
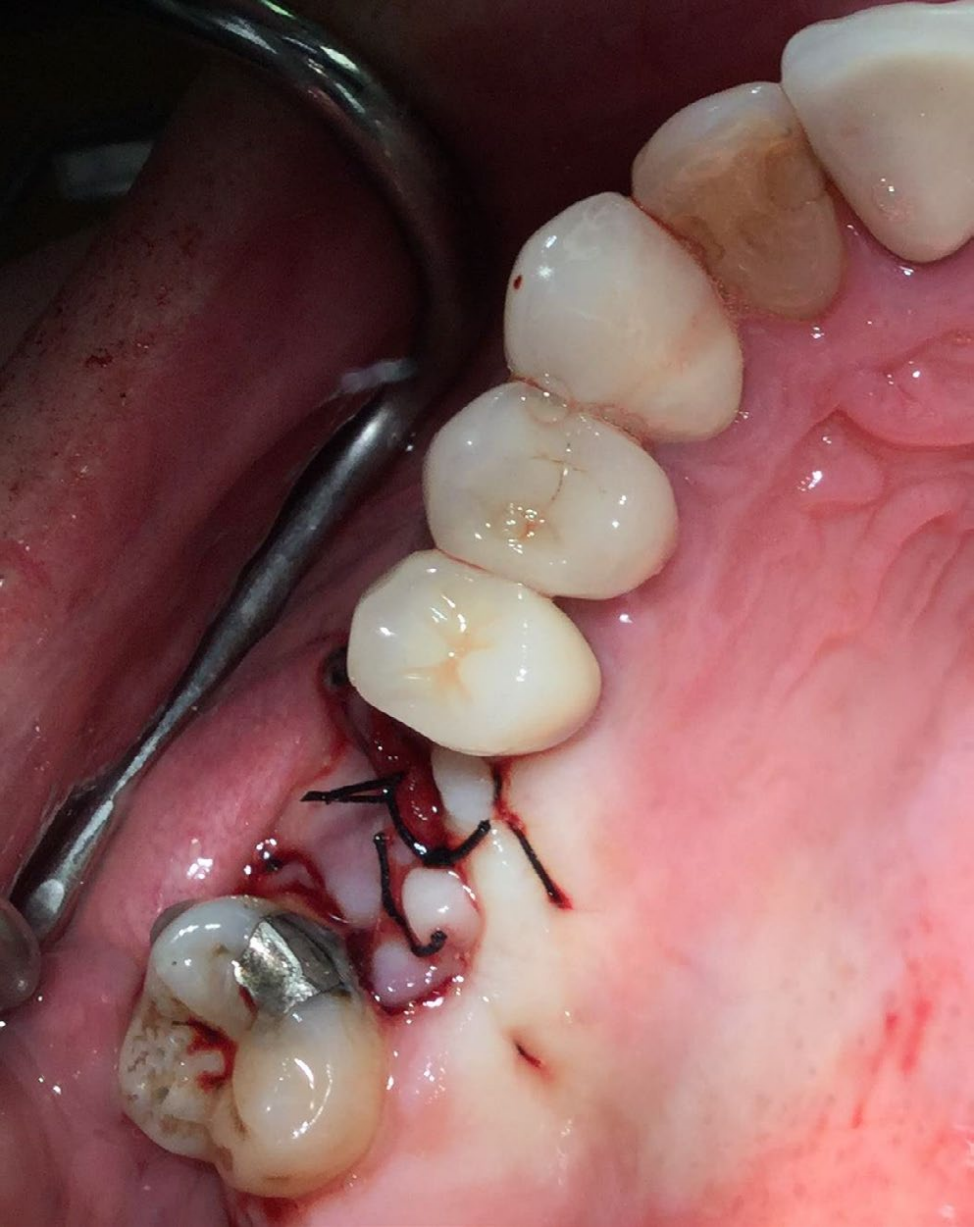
A buccal fat pad pedicle flap was made and primarily closed with 3‐0 black silk suture

**FIGURE 5:**
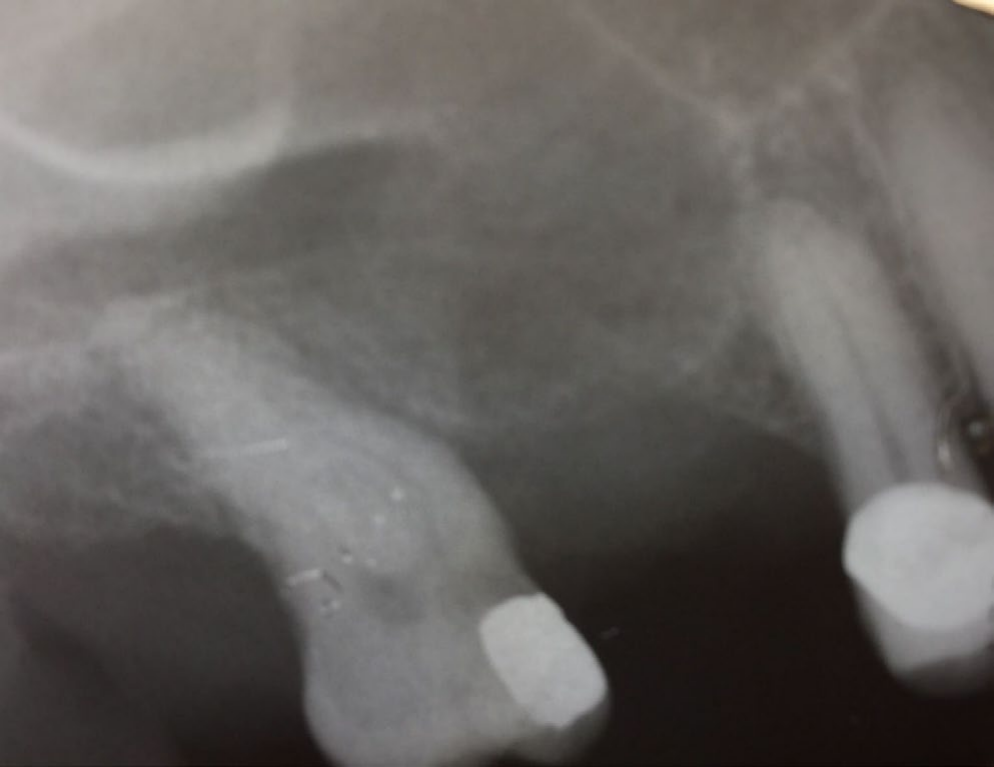
Radiograph of the treated site

The protruding soft lining was carefully cleaned with chlorhexidine (Peridex) and rinsed with saline. The prolapsed lining was pushed superiorly into the socket to the perceived level of the sinus floor and held with two collagen plugs. A buccal fat pad pedicle flap was made and held with mattress technique 3‐0 black silk sutures for primary closure (Figures 4 and 5). Sinus care instructions were given that included no nose blowing and proper sneezing technique.

The patient returned in 10 days for suture removal. There was no pain, signs of infection, or oral‐antral communication.

After 5 weeks at follow‐up, the patient reported sensing a small perforation at the wound site. There was no air flow communication. This was monitored in hopes of a spontaneous complete closure. Oral amoxicillin clavulanic acid (Augmentin, Sandoz) was instituted for 1 week along with an over‐the‐counter sinus decongestant nasal spray, oxymetazoline (Afrin), and instructions.

**FIGURE 6:**
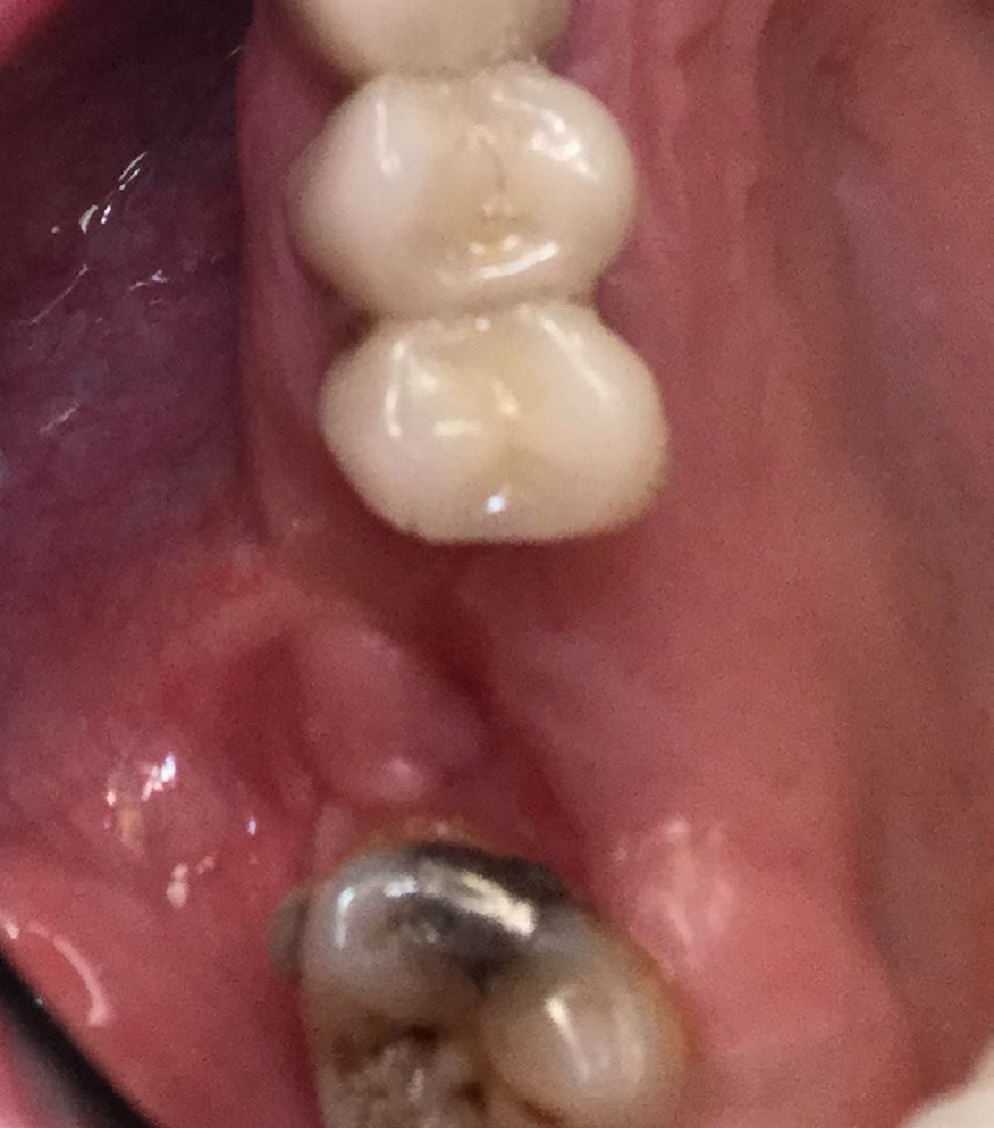
The patient was followed for several weeks and healed uneventfully

The patient was followed for 3 additional weeks. The site healed uneventfully with no oral‐antral communication or fistula (Figure 6). After 10 postoperative weeks, there has been no occurrence of an oral‐antral communication or prolapse.

Caries was subsequently treated, and the patient prescribed a fluoridated oral daily rinse (0.2% neutral sodium fluoride, Prevident Rinse, Colgate).

## 3 DISCUSSION

After a molar tooth extraction, there can be a perforation of the lining of the maxillary sinus. Many, if not most, of these heal uneventfully with no surgical intervention.^3^ In the case reported herein, the sinus lining was not perforated, yet the lining apparently herniated into the socket by respiratory air pressure. Air flow in the sinuses is not well understood, and partial closure of an ostium may increase sinus pressure.^3^

An inflamed sinus lining may impede or close the ostium. This may increase sinus pressure that could induce the expansion of the lining into the oral cavity. Thus, a sinus decongestant may reduce the likelihood of such an occurrence.

The sinus lining or mucosa is essentially an epithelial (pseudostratified ciliated columnar epithelium with goblet cells and seromucinous glands) balloon supported by osseous walls. If the sinus mucosal support is lost due to an extraction, then the lining is unsupported and respiration pressure pushes on the unsupported lining. Empirically, any loss of the respiratory epithelial attachment to the supporting bone, such as by an extraction, may allow a detachment of the lining that may allow a “ballooning” or expansion of the lining through the extraction socket into the oral cavity. This apparently took 4 weeks to occur and was enough time for the epithelial cells to proliferate or for the tissue to stretch, creating a prolapse of the lining. This herniation may have been a polyp, a mucocele or a simple expansion of the sinus lining.

### 3.1 Polyps

Respiratory epithelial polyps are prolapsed mucosal lesions that arise from the mucosal lining of the paranasal sinuses and lateral walls of the nasal cavity. Etiology is multifactorial involving the inflammatory response of the mucosal lining.^4^ These generally require no treatment.

A herniation prolapse of the sinus lining may be an antral polyp.^2^ Takeda reported a herniation of the sinus lining through an oroantral fistula that appeared as a polypoid lesion on the alveolar ridge.^2^ In this reported case, the maxillary molar had been extracted 2 months prior. There were no symptoms or signs of infection. It appeared as a red nontender soft‐tissue mass on the alveolar ridge. The prolapse was repositioned and covered with a pedicle graft and healed well.^2^ The sinus lining prolapse was described by Takeda as a “red” bulge.^2^ The lesion reported now herein had a red and yellow appearance which may have been the result of a remnant of an apical lesion from a less than perfect debridement or discolorization from sinus or oral fluids. Since no biopsy was performed on this or any previously reported case, there is no definitive evidence as to the histology of these lesions.

Maxillary sinusitis infections with a specific bacterium such as Streptococcus pneumoniae, Bacteroides fragilis, and Staphylococcus aureus may not be direct causes of sinus polyp formation.^5^ Polyps develop after mucosal inflammation that induces fibroblast proliferation, angiogenesis, and epithelial migration to cover the polyp. Polyps can be initiated by any number of stimuli. Polyp formation is the result of continuous inflammation and is not directly related to any specific microorganism. Nonetheless, a virulent microorganism that induces deep mucosal inflammation may initiate polyp formation.^5^, ^6^ Thus, a residing bacterial species in a sinus lining polyp may be a contributing cause but probably not a major culprit unless the species is particularly virulent.^5^, ^6^

Advanced periodontitis with extension through the alveolar process extending into the antrum may initiate mucosal thickening and polyp formation.^5^, ^6^ Inflammatory infiltration, edema, fibrosis of the tunica propria, mucous‐serous gland proliferation, interstitial pseudocyst formation, polyp formation, hyalinization of the connective tissue lining, thrombosis of blood vessels, and metaplastic and degenerative changes can occur in the epithelial lining. There may be a direct relationship between moderate and severe periodontitis of the maxillary molar teeth and mucosal thickening of the maxillary sinus.^6^ Although the prolapse patient reported herein did not have a severe periodontitis, he did indeed have a history of dental pulpal infections which may have extended into the antrum. Nonetheless, the prolapse lesion was probably not a polyp.

### 3.2 Mucocele

A mucocele is a cystic lesion that results from an obstruction of a draining ostia.^6^, ^7^ Most sinus mucoceles occur in the frontal and ethmoidal sinuses. A mucocele that herniates into the submucosa is an internal mucocele, while one that herniates through the bone wall is an external mucocele. Expansion of a mucocele occurs in the direction of least resistance. A sinus drainage block may result in mucocele formation. Histologically, an external sinus mucocele may have flattened pseudostratified ciliated columnar epithelium.^6^, ^7^ The pro‐operative and postoperative periapical radiographs in the case reported herein do appear to be somewhat opacified which may indicate a blocked ostium.^6^, ^7^ A CBCT may demonstrate sinus pathologies better than plane film radiographs.^8^

Infected teeth can produce antral mucoceles.^9^, ^10^, ^11^ Treatment may entail extraction of the tooth and lesion excision. Maxillary odontogenic infections may cause up to 12% of all sinusitis cases.^10^, ^11^

Intercellular adherence of the sinus epithelial cells is loose and very permeable which may allow stretching of the lining and prolapse through an unsupported lining.^12^ Thus, the prolapse lining may have been a mucocele induced by an impaired ostium.

### 3.3 Decongestants

Disorders of human respiratory function are very common and may become a chronic part of a patient's life that becomes unnoticed.^12^ Some people use nasal sprays and systemic antihistamines routinely.

Oxymetazoline acts by constricting nasal blood vessels to reduce swelling and congestion.^12^ It can exacerbate benign prostate hypertrophy, heart failure, and diabetes. Thus, overuse may complicate these conditions.^12^

Preoperative or postoperative decongestants may be appropriate for prevention of sinus lining herniations and aid in sinus communication closures.

Optimal clearance of the ciliated sinus epithelium is best done at a body temperature of 37 degrees C and 100% humidity.^12^ Diabetics of more than 10 years may have attenuated clearance ability of the ciliated sinus lining.^12^

The decongestant preservative, benzalkonium chloride, can eradicate microorganisms but also reduce the effectiveness of sinus phagocytosis.^12^ Thus, this preservative should be avoided.

Sudden discontinuance of a decongestant may induce rebound swelling and impair air flow.^12^

### 3.4 Surgical closure

The site reported herein was primarily closed with a buccal fat pad pedicle graft. The buccal fat pad pedicle graft may be the most reliable technique for sinus closure.^13^, ^14^

Normal anatomical variations may cause some patients to be susceptible to sino‐nasal disorders and complicate reparative surgery.^15^ The sinus ostium a can be surgically enlarged to reduce symptoms and increase clearance.^16^

## 4 CONCLUSIONS

It may be possible for an intact sinus lining to prolapse or herniate into the oral cavity through a recent maxillary molar tooth extraction socket. Such a herniation or prolapse may be an external mucocele caused by a sinusitis with blockage of the ostium. The mucocele may form due to a blocked or impaired sinus ostium that creates sinus pressure causing the sinus lining to protrude through the extraction socket. Thus, patients with a blocked sinus ostium may be prone to herniation of the sinus lining through the extraction socket. Any postoperative instructions for maxillary molar extraction should include sinus care for nose blowing and sneezing.

**Clinical relevance**

*Rationale*: A herniation of the sinus lining into the oral cavity is a rare finding but may develop into a fistula.

*Principle finding*: A prolapse of the sinus lining can be corrected with a buccal fat pad pedicle graft.

*Practical implications*: Patients with a blocked sinus ostium may be prone to protrusion of the sinus lining. Postoperative instructions should include caution for nose blowing and sneezing and use of a sinus decongestant.

## CONFLICT OF INTEREST

There are no conflicts of interest, commercially or political or otherwise.
